# Physicochemical Properties of the Mammalian Molecular Chaperone HSP60

**DOI:** 10.3390/ijms19020489

**Published:** 2018-02-06

**Authors:** Ryuichi Ishida, Tomoya Okamoto, Fumihiro Motojima, Hiroshi Kubota, Hiroki Takahashi, Masako Tanabe, Toshihiko Oka, Akira Kitamura, Masataka Kinjo, Masasuke Yoshida, Michiro Otaka, Ewa Grave, Hideaki Itoh

**Affiliations:** 1 Department of Life Science, Graduate School and Faculty of Engineering Science, Akita University, Akita 010-8502, Japan; i4d@sanken.osaka-u.ac.jp (R.I.); okamoto-tomoya@ichimaru.co.jp (T.O.); hkubota@gipc.akita-u.ac.jp (H.K.); a-u.t-hiroki@med.akita-u.ac.jp (H.T.); m-ishida@biken.osaka-uac.jp (M.T.); grave@gipc.akita-u.ac.jp (E.G.); 2 Department of Molecular Biosciences, Faculty of Life Sciences, Kyoto Sangyo University, Kamigamo, Kita-ku, Kyoto 803-8555, Japan; fmotojim@gmail.com (F.M.); masasuke.yoshida@cc.kyoto-su.ac.jp (M.Y.); 3 Department of Physics, Faculty of Science, Shizuoka University, 836 Ohya, Suruga, Shizuoka 422-8529, Japan; stoka@ipc.shizuoka.ac.jp; 4 Laboratory of Molecular Cell Dynamics, Faculty of Advanced Life Science, Hokkaido University, N21W11, Kita-ku, Sapporo 001-0021, Japan; a_kita@mail.sci.hokudai.ac.jp (A.K.); kinjo@sci.hokudai.ac.jp (M.K.); 5 Department of Gastroenterology, Juntendo University School of Medicine, Bunkyo-Ku, Tokyo 113-8421, Japan; mootaka@juntendo.ac.jp

**Keywords:** molecular chaperone, chaperonin, HSP60, GroEL

## Abstract

The *E. coli* GroEL/GroES chaperonin complex acts as a folding cage by producing a bullet-like asymmetric complex, and GroEL exists as double rings regardless of the presence of adenosine triphosphate (ATP). Its mammalian chaperonin homolog, heat shock protein, HSP60, and co-chaperonin, HSP10, play an essential role in protein folding by capturing unfolded proteins in the HSP60/HSP10 complex. However, the structural transition in ATPase-dependent reaction cycle has remained unclear. We found nucleotide-dependent association and dissociation of the HSP60/HSP10 complex using various analytical techniques under near physiological conditions. Our results showed that HSP60 exist as a significant number of double-ring complexes (football- and bullet-type complexes) and a small number of single-ring complexes in the presence of ATP and HSP10. HSP10 binds to HSP60 in the presence of ATP, which increased the HSP60 double-ring formation. After ATP is hydrolyzed to Adenosine diphosphate (ADP), HSP60 released the HSP10 and the dissociation of the double-ring to single-rings occurred. These results indicated that HSP60/HSP10 undergoes an ATP-dependent transition between the single- and double-rings in their system that is highly distinctive from the GroEL/GroES system particularly in the manner of complex formation and the roles of ATP binding and hydrolysis in the reaction cycle.

## 1. Introduction

Molecular chaperones assist in the folding of proteins [1,2]. Chaperonins are a family of molecular chaperones that share a double ring-like structure and play an essential role in the folding of newly synthesized and denatured proteins [3,4]. The group I chaperonin, *Escherichia coli* GroEL have been well studied, particularly for their structure and function. GroEL is an approximate 57 kDa protein and has a cylindrical tetradecamer structure composed of two back-to-back rings, each of which contains seven subunits [5,6]. Adenosine triphosphate (ATP) binding to the GroEL induces the formation of GroEL/GroES complex to form a cavity for encapsulation of substrates. After the hydrolysis of ATP, GroES dissociates from GroEL and the capsulated substrate is released. This cycle is repeated ATPase-dependently.

In contrast to the well-studied nature of GroEL/GroES complexes, the molecular structure and function of the chaperonin homolog, heat shock protein, HSP60/HSP10, has remained unclear. HSP60 plays an essential role in assisting the folding of imported proteins and refolding denatured proteins in the mitochondria [7,8,9,10,11,12]. HSP60 has a signal sequence in its N-terminus (1–26 amino acid residues). Therefore, it has been thought to be transported into the mitochondria by a process similar to that of other imported mitochondrial proteins [13,14]. After transport to the mitochondria, HSP60 is converted into a mature form of lower molecular mass (58 kDa) [14,15] and then exists in equilibrium between monomers, single rings and double rings in the presence of ATP [16,17,18,19]. We also reported that native HSP60 and HSP10 purified from porcine formed football-type and bullet-type complexes in the presence of ATP [20]. Unlike the GroEL/GroES system, the affinity between HSP60 and HSP10 in the presence of adenosine diphosphate (ADP) is very low [17] and the inhibition of the HSP60/HSP10 system folding and ATPase activity by ADP is weak. Recently, the crystal structure of human HSP60/HSP10 complex was revealed by using HSP60 (E321K) mutant which has an open apical region and high affinity with co-chaperones, which showed the difference in the inter-ring arrangement and nucleotide condition in the complex with HSP10 [21,22]. These results suggest that there are differences between the GroEL/GroES and HSP60/HSP10 systems. However, how the nucleotides regulate the various states of HSP60, including the double- and single-rings and monomer, has remained unclear. We have now analyzed the nucleotide- and its co-chaperon-dependent transition between the various states of HSP60 by transmission electron microscopic observations, small angle X-ray scattering (SAXS), fluorescence cross-correlation spectroscopy (FCCS) and other biochemical methods. We found that HSP60 forms double-ring structures from single rings in an ATP-dependent manner and that HSP60/HSP10 stably forms football-type complexes in the presence of ATP. Their characteristics are highly distinctive from those of the stable double-ring GroEL, suggesting that the complex formation pathway of HSP60/HSP10 is specific to mitochondria.

## 2. Results

### 2.1. The Double Ring Formation of HSP60 Depends on the Nucleotide Conditions and HSP10

**Figure 1:**
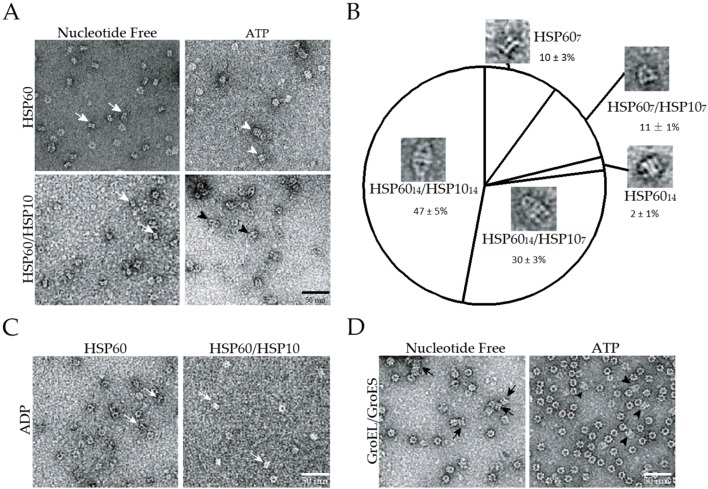
Transmission electron microscopy observation of chaperonin complex. (**A**) Transmission electron microscopic analysis of heat shock protein, HSP60 (upper) or HSP60/HSP10 (lower) in the presence (right) or absence (left) of 1 mM ATP. White arrows, white arrowheads and black arrowheads indicate side views of single rings (HSP60_7_), double rings (HSP60_14_) and football-type complexes (HSP60_14_-HSP10_14_), respectively; (**B**) Classification of five shapes of HSP60/HSP10 complex observed in the presence of 1 mM adenosine triphosphate (ATP). More than 500 side views of HSP60 complexes were counted for each experiment. Mean and standard deviations of three independent experiments are shown; (**C**) HSP60 alone (left) or HSP60/HSP10 (right) in the presence of 1 mM adenosine diphosphate (ADP). White arrows indicate side views of single rings (HSP60_7_). (HSP10/single ring HSP60 ratio = 2); (**D**) GroEL with GroES in the presence (right) and absence (left) of 1 mM ATP. Black arrows and arrowheads indicate double rings (GroEL_14_) and bullet-type (GroEL_14_-GroES_7_), respectively. (GroES/double ring GroEL ratio = 4).

To analyze the molecular size and structure of HSP60 in the presence or absence of the co-chaperone HSP10, we purified human recombinant HSP60 and HSP10, as well as GroEL and GroES as a controls (Figure S1A). The native-poly acrylamide gel electrophoresis (PAGE) analysis of HSP60 and GroEL indicated that HSP60 migrates more rapidly than the 14-mer double ring of GroEL in native gels (Figure S1B). Size exclusion chromatography with molecular size standards indicated that the molecular size of the human HSP60 is approximately 426 kDa (Figure S1C). Based on the fact that the molecular weight of the HSP60 monomer is 58.1 kDa, the HSP60 complex is estimated to contain 7.3 subunits. In contrast, the molecular size of GroEL was estimated to be 791 kDa, corresponding to 13.8 subunits. Moreover, a transmission electron microscopic analysis of HSP60 indicated that side views are very similar to those of GroEL-SR1, a heptamer single-ring mutant of GroEL (Figure 1A, upper-left) [23]. Top views of HSP60 showed seven-fold symmetry rings. These results clearly indicate that HSP60 exists as a stable heptamer single ring in the absence of ATP. To analyze the effects of HSP10 and ATP-dependent double-ring formation of HSP60, we analyzed the structure of the HSP60 complex in the presence of HSP10. HSP60 was unable to associate with HSP10 in the absence of ATP and remained as single rings (Figure 1A, lower-left). The addition of ATP induced the association of HSP10 with HSP60. Side views of HSP60-containing molecules were divided into five categories: HSP60_14_-HSP10_14_ (football-type, 47%), HSP60_14_-HSP10_7_ (bullet-type, 30%), HSP60_14_ (double ring alone, 2%), HSP60_7_-HSP10_7_ (single ring with a lid, 11%) and HSP60_7_ (single ring alone, 10%) (Figure 1A, lower right, and Figure 1B). These results indicated that approximately 80% of the HSP60-containing complexes possess the HSP60 double-ring structure and suggest that the ATP-dependent HSP60 double ring is stabilized by HSP10 binding. Interestingly, these football-type complexes were more frequently found than the bullet-type complexes. Unlike the HSP60/HSP10 complexes, all side views of the GroEL/GroES complex were bullet types (Figure 1C). These results indicated that HSP60/HSP10 forms the football-type complex much more easily than does GroEL/GroES. It is noteworthy that HSP60 does not form double-rings nor associates with HSP10 in the presence of ADP (Figure 1D), although GroEL is able to associate with GroES in the presence of these nucleotides [6,24,25].

### 2.2. The Association of the HSP60 Single Rings Was Induced by the Presence of ATP and Increased by the Addition of HSP10

**Figure 2:**
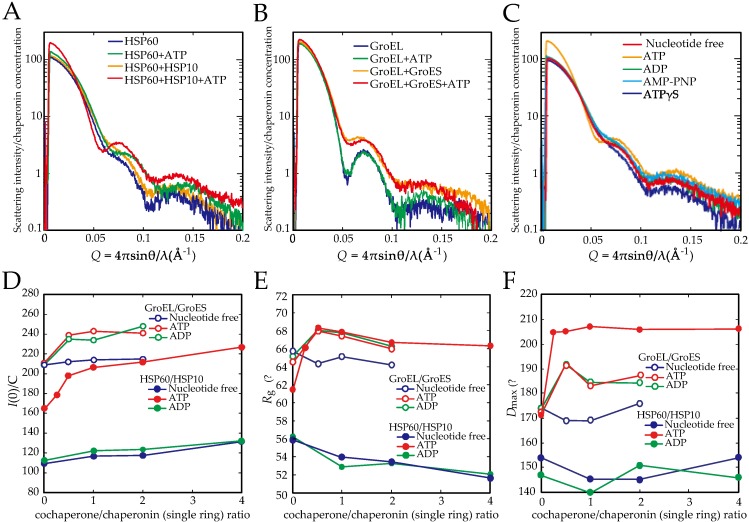
Small angle X-ray scattering (SAXS) analysis of HSP60/HSP10 and GroEL/GroES complex under the various nucleotide conditions. (**A**,**B**) SAXS patterns of HSP60 and GroEL under various conditions. Scattering intensities of HSP60 (**A**) and GroEL (**B**) with or without their co-chaperones in the presence and absence of 1mM ATP. Co-chaperone (single ring)/chaperonin (as double ring) ratio = 2; (**C**) SAXS patterns of HSP60/HSP10 in the presence of various nucleotides including ATP, ADP, Adenosine-5′-(β, γ-imido)-triphosphate (AMP-PNP) and Adenosine 5′-(γ-thio)-triphosphate (ATPγS); (**D**) *I*(0)/C values of HSP60 (solid circle) and GroEL (open circle) in the presence of various concentrations of co-chaperones under the nucleotide-free (Blue), 1 mM ATP (Red) or ADP (Green) conditions; (**E**) *R*_g_ values of HSP60 (solid circle) and GroEL (open circle) in the presence of various concentrations of co-chaperones under the conditions indicated were analyzed by a Guinier plot. Protein concentrations were 3 mg/mL HSP60 and 0.15–2.25 mg/mL HSP10, or 3 mg/mL GroEL and 0.15–2.25 mg/mL GroES when cochaperone/chaperonin (single ring) ratio = 1:1; (**F**) *I*(0)/C D_max_ values of HSP60 (solid circle) and GroEL (open circle) in the presence of various concentrations of co-chaperones under the nucleotide-free (Blue), 1 mM ATP (Red) or ADP (Green) conditions.

To analyze the HSP60 double-ring formation and HSP60/HSP10 complex formation under more physiological conditions, we analyzed these complexes and GroEL/GroES complexes as a control in solution by SAXS (Figure 2A,B). The X-ray scattering patterns of HSP60 in the absence of nucleotides indicated that the addition of HSP10 had no effect on the overall structure (Figure 2A). In contrast, a shoulder was observed at 0.08 Å^−1^ in the presence of ATP (Figure 2A, green line), and the shoulder shifted to an obvious peak by the addition of HSP10 (Figure 2A, red line). Other nucleotides (ADP, adenosine 5′-(γ-thio)-triphosphate (ATPγS) and adenosine-5′-(β, γ-imido)-triphosphate (AMP-PNP)) had no effect on the scattering pattern of HSP60 (Figure 2C). These results suggested that ATPγS and AMP-PNP did not act as ATP analogs for human HSP60. These scattering patterns indicated that the molecular size and shape of HSP60 were changed by the addition of ATP and HSP10. Guinier analysis of the scattering intensity was performed to calculate *I*(0)/C, which is the forward scattering intensity standardized by the protein concentration of HSP60 or GroEL (Figure 2D). In the absence of nucleotides, the *I*(0)/C value for HSP60 was approximately half the value for GroEL, indicating that the molecular size of HSP60 is half that of GroEL in solution. These results support the notion that HSP60 exists as single rings in the absence of ATP. The addition of ATP increased the *I*(0)/C value 1.5-fold in the absence of HSP10 (at HSP10/HSP60 ratio = 0), suggesting that the double-ring HSP60 content was 50%. Moreover, the addition of HSP10 and ATP to HSP60 further increased the *I*(0)/C value, and HSP60-containing molecules were estimated to be mostly double rings in the presence of HSP10 (at HSP10/HSP60 ratio = 1). On the other hand, HSP10 and ADP did not significantly increase the *I*(0)/C value of HSP60 as well as the absence of nucleotides although the addition of GroES and ADP increased the value of GroEL. These results indicated that ADP does not stimulate the double-ring formation and the interaction with HSP10 unlike the GroEL/GroES system. Similar results were obtained for the calculated radius of gyration (*R*_g_) values (Figure 2E), although these values gradually decreased beyond the peaks at the co-chaperone/chaperone single-ring ratio of 0.5, probably due to the presence of an increasing amount of free co-chaperones.

To obtain the maximum particle distance (*D*_max_), which correlates with the maximum length in the various molecules, pair distribution P(r) functions were obtained after the scattering patterns were analyzed by an indirect Fourier transformation method using the GNOM software package [26] (Figure S2). The addition of GroES slightly (1.1-fold) increased the *D*_max_ value of GroEL in the presence and absence of ATP, probably indicating the formation of bullet-type complexes (Figure 2F). The value of HSP60 was calculated as half that of GroEL, indicating that HSP60 is a single-ring. The addition of HSP10 has no effect on this value in the ADP or nucleotide-free conditions. The addition of ATP increased the value of HSP60 to that of GroEL, indicating that a double-ring formation of HSP60 was induced by ATP. Moreover, the addition of HSP10 and ATP increased the value of HSP60 more than that of GroEL/GroES bullet complex. Since the value of HSP60/HSP10 complex is similar to that of GroEL with two GroES, this result suggested that the HSP60/HSP10 complex forms the football complex.

We also calculated the curve fitting of the scattering patterns by theoretical models (Figure S2). Although the curve fittings at a lower *Q* value were fair, those were deviated at >0.06 Å^−1^. These discrepancies would be attributed to the difference of solution structures from the crystal structures. Therefore, the estimation of the population of HSP60 complexes from SAXS patterns would be unreliable. The fitting estimated that single-ring and double-ring HSP60 in HSP60+ATP existed at the ratio of 0.86 and 0.14. The estimated content of double-ring was lower than the value estimated from *I*(0)/C (50%). In the presence of HSP10 and ATP, the scattering pattern was largely changed, and the curve fitting estimated that single-ring HSP60, single-ring HSP60/HSP10 complex, and the football complex existed at the ratio of 0.09, 0.57, and 0.34, respectively. This ratio is not largely different from the population observed in electron microscopy analysis (Figure 1)

### 2.3. The Association between HSP60 and HSP10 Was Induced by Only ATP

**Figure 3:**
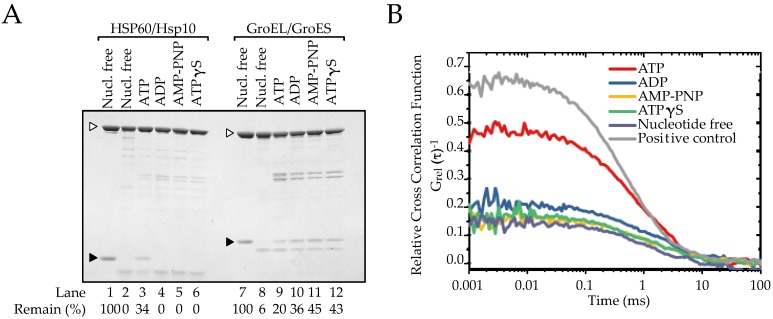
Association of HSP10 to HSP60 under various nucleotide conditions. (**A**) Trypsin resistance of HSP60/HSP10 or GroEL/GroES under nucleotide-free, 1 mM ATP, ADP, AMP-PNP or ATPγS condition. Digested samples were analyzed by SDS-PAGE (12% gels). White and black arrowheads indicate HSP60/GroEL and HSP10/GroES positions, respectively; (**B**) FCCS analysis of the association between HSP60 and HSP10. Cross-correlation functions of Atto 647N-labeled HSP60 and Atto 488-labeled HSP10 under nucleotide-free (dark grey), 1 mM ATP (red), ADP (blue), AMP-PNP (yellow), ATPγS (green) condition, or both Atto 488- and Atto 647N-labeled HSP60 as a positive control (light grey) are indicated.

To measure the stability of interaction between the HSP60 and HSP10, a protease sensitivity assay was performed at a co-chaperone/chaperonin single-ring ratio of 1.0. Although HSP10 was digested by trypsin in the absence of nucleotides (Figure 3A, lane 2), approximately 40% of HSP10 showed resistance to trypsin digestion in the presence of ATP (Figure 3A lane 3). However, other nucleotides (ADP, ATPγS and AMP-PNP) had no effect on the resistance (Figure 3A, lanes 4–6). These results suggest that HSP10 associates with HSP60 in the presence of ATP but not in the presence of ADP nor the non-hydrolyzable analogues of ATP. This result is different from the case of porcine HSP60/HSP10 which associated with HSP10 by ATP or AMP-PNP [20]. The same protease sensitivity assay was performed using GroEL/GroES. Although GroES was digested in the absence of nucleotide, 20% of GroES became resistant to protease digestion in the presence of ATP (Figure 3A, lanes 9). In contrast to HSP60/HSP10, GroES became resistant (~50%) to protease digestion in the presence of ADP, ATPγS and AMP-PNP (Figure 3A, lanes 10–12). These results are consistent with previous observations that GroEL associates with GroES and forms a bullet-type complex in the presence of these nucleotides. These results indicated that the nucleotide conditions required for the binding with the co-chaperone are different between HSP60/HSP10 and GroEL/GroES.

Moreover, to determine the dissociation constant (*K*_d_) between HSP60 and HSP10 rings during dynamic interaction in solution, we employed a fluorescence cross correlation spectroscopy (FCCS) analysis in the presence of various nucleotides. In this method, molecules are labeled by two different fluorescent dyes and the number of interacting molecules in the total fluorescent molecules is analyzed by a confocal optical system with a single molecule sensitivity [27,28,29]. HSP60 and HSP10-A2C (a mutant prepared for fluorescent dye labeling) were labeled with Atto 647N and Atto 488, respectively. We confirmed the association of the fluorescently labeled HSP10-A2C with the labeled HSP60 by protease sensitivity assay (Figure S3). The auto-correlation functions and cross-correlation function were calculated from the recording of fluorescence fluctuations (Figure S4). The addition of ATP induced elevation of the cross-correlation function (Figure 3B). In contrast, cross-correlation curves were only slightly affected in the presence of ADP, ATPγS or AMP-PNP (Figure 3B). The content of the HSP60/HSP10 complexes in the total HSP60-containing particles under these conditions was calculated as relative cross correlation amplitude (RCA), which indicated the relative strength of interaction (Table 1). To estimate the binding ratios of HSP60 and HSP10, the observed RCA values were standardized against the maximum RCA value determined by using double-labeled HSP60 as a positive control and a mixture of two dyes as negative control (Figure S5). The HSP60-HSP10 binding ratio in the presence of ATP was 86.8%, and this value was dramatically higher than that in the presence of the other nucleotides or the absence of nucleotides. In the absence of nucleotides, the *K*_d_ value was 7.5 × 10^−7^ M, and similar values were obtained in the presence of ATPγS or AMP-PNP. The *K*_d_ value slightly decreased only in the presence of ADP (5.1 × 10^−7^ M). In contrast, the *K*_d_ value in the presence of ATP (8.7 × 10^−8^ M) was dramatically lower than those under the other conditions. Taken together, these observations suggest that HSP60 becomes competent to bind to HSP10 in the presence of ATP but not in the presence of ADP or the non-hydrolyzable analogues of ATP.

### 2.4. ATP Binding State of HSP60 Stably Interacts with HSP10 and Forms a Football-Type Complex

**Figure 4:**
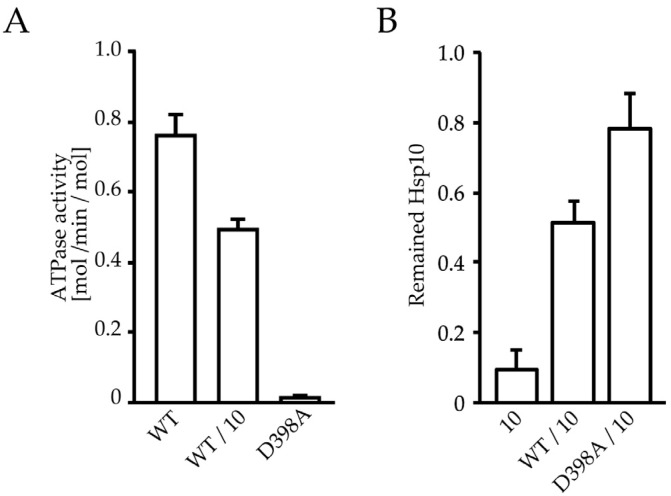

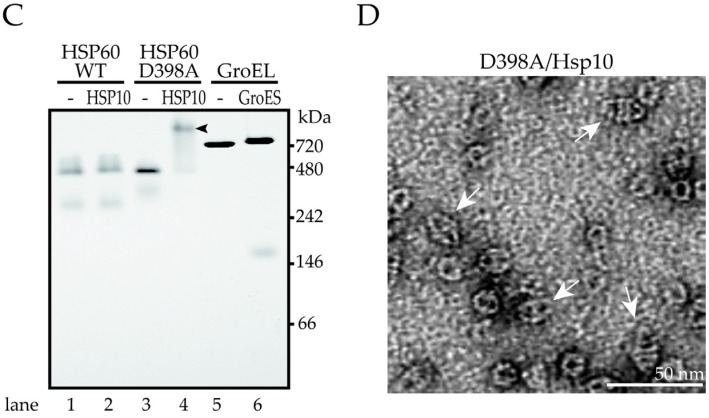
The formation of stable football complex of HSP60(D398A)/HSP10/ATP. (**A**) ATPase activity of wild type HSP60, HSP60/HSP10 or D398A mutant of HSP60 (0.1 μM). (**B**) Protease sensitivity assay of HSP10 without HSP60, with wild-type HSP60 or HSP60(D398A) in the presence of 1 mM ATP. The values were quantified from stained bands of HSP10 in SDS-PAGE gel. (**C**) Native-PAGE analysis of wild type HSP60, HSP60(D398A), or GroEL with or without their co-chaperone in the presence of ATP. Native-PAGE gel and buffer were supplemented with 1 mM ATP. The arrow indicates the putative complex of HSP60(D398A)/HSP10/ATP. (**D**) Electron microscopic analysis of HSP60(D398A)/HSP10 in the presence of ATP. White arrows indicate side views of HSP60(D398A)/HSP10 football-type complexes.

Our results suggest that HSP60 is dynamically exchanged between the single-ring and double-ring during an ATPase-dependent reaction cycle, but not in the presence of ADP (Figure 1 and Figure 2). HSP10 increased the double ring formation of HSP60 although a part of HSP60 constantly exists as a single ring even in the presence of ATP (Figure 1 and Figure 2). The ATPase activity of HSP60 decreased by HSP10 as in the GroEL/GroES system (Figure 4A) [17]. Therefore, the ATP binding state of HSP60 was thought to stabilize the HSP60 double ring formation. To verify this notion, we prepared a D398A mutant of HSP60. The D398A mutant of GroEL was defective in the ATP hydrolysis (approximately 2% of wild-type ATPase activity) although this mutant binds ATP with a normal affinity and forms a football complex with GroES [30,31]. ATPase activity of HSP60(D398A) was also defected (Figure 4A). The protease digestion assay of HSP10 indicated that HSP10 stably associated with HSP60(D398A) more than wild-type HSP60 (Figure 4B). The oligomeric state of HSP60 was analyzed by native-PAGE in the presence of ATP (Figure 4C). Wild-type HSP60 migrated as single-ring even in the presence of ATP and HSP10 (Figure 4C, lane 1 and 2). Although HSP60(D398A) also migrated as a single-ring in the presence of ATP, HSP60(D398A) migrated as a double-ring in the presence of ATP and HSP10 (Figure 4C, lane 4). These results suggested that the association of single-rings of wild-type HSP60 was relatively weak and then the double-ring tends to separate to a single-ring during native-PAGE. Electron microscopic observation of HSP60(D398A) showed that most of them readily formed football-type complexes with HSP10 in the presence of ATP without forming a single-ring (Figure 4D). These results suggested that the ATP binding state of HSP60 stably interacts with HSP10 and forms the football-type complex.

### 2.5. HSP60/HSP10 Complex Dissociates after ATP Was Hydrolyzed

**Figure 5:**
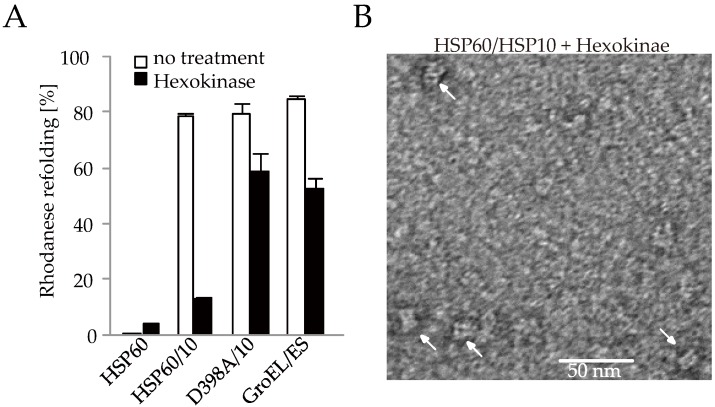
Rhodanese refolding assay to measure the folding cavity formation of chaperonin. (**A**) Rhodanese activity by chaperonins after 60 min (*n* = 3). HSP60/HSP10, HSP60(D398A)/HSP10, and GroEL/GroES are used. Hexokinase was added at 3 s after the initiation of the folding reaction. The time course of rhodanere refolding was shown in Figure S6. (**B**) Electron microscopic analysis of HSP60/HSP10 complex after hexokinase treatment. White arrows indicate side views of HSP60 single-ring.

To evaluate whether the double-ring HSP60/HSP10 complex dissociates after ATP hydrolysis, we analyzed the formation of the HSP60/HSP10-folding cavity by monitoring the substrate folding because the folding activity is lost by the dissociation of HSP10 from HSP60. In the case of GroEL/GroES, it was reported that the hydrolysis of excess ATP to ADP by hexokinase immediately after the initiation of the folding reaction inhibited subsequent folding reaction cycles but bullet-type GroES/GroES/ADP was stably formed and 50~60% of total rhodanese was folded in the closed cavity (Figure 5A and Figure S6) [31,32]. The denatured rhodanese was refolded by HSP60/HSP10 (Figure 5A). However, this folding reaction was largely inhibited by hexokinase treatment (Figure 5A). Only single-ring of HSP60 was observed after hexokinase treatment by transmission electron microscopy (Figure 5B). These results indicated that HSP10 was released from HSP60 and double-ring of HSP60 dissociates and turns to single-ring after ATP hydrolysis. In the case of HSP60(D398A), its refolding activity was similar to that of wild-type HSP60/HSP10 but hexokinase treatment did not largely inhibit rhodanese refolding (Figure 5A). This result indicated that the football complex of HSP60(D398A) remained in an ATP-bound state even after hexokinase treatment and thus most of rhodanese was folded in the two closed chaperonin cavities. Taken together, these observations strongly suggest that HSP60 forms a complex with HSP10 at the ATP-bound state and dissociates into a single-ring after the bound ATP was hydrolyzed.

## 3. Discussion

In this study, we elucidated the conditions required to form football-type, double-ring, and single-ring complexes. It has been reported that the dissociation from single-ring HSP60 to monomer was observed by size exclusion chromatography [18]. However, such dissociation was not observed in our size exclusion chromatography and SAXS analysis (Figures S1 and S2). The single-ring, double-ring, bullet-type, and football-type complexes were observed as previously reported [16,17,18,19,20]. In this study, we focused on the population of these complexes and the condition for their formation. About half of HSP60 was observed as a double-ring in the presence of ATP but no double-ring was detected in the presence of ADP or non-hydrolysable ATP analogs (Figure 2 and Figure 3). The addition of HSP10 to HSP60/ATP facilitated double-ring formation including football-type and bullet-type complexes (~70%) (Figure 1). The ATPase-deficient mutation of D398A stabilized the formation of the football-type complex in the presence of HSP10, but not in the absence of HSP10 (Figure 4). In contrast to ATP, ADP did not induce the double-ring formation even in the presence of HSP10 as reported [17]. Recently, Nisemblat and coworker succeeded to crystallize the football complex of HSP60/HSP10 using the HSP60(E321K) mutant which has an open apical domain [21]. This mutant associates with HSP10 and forms double-rings in the presence of ADP though wild type (WT) HSP60 did not, as shown in our study. The crystal structure showed that some residues at the interface between rings contribute to double-ring formation. After ATP hydrolysis in the equatorial domain of HSP60 and subsequent release of HSP10, the equatorial domain changes its conformation and these interactions would be lost. Therefore, not only did ATP induce conformational change of HSP60, it also induced stabilization of the conformation by HSP10 binding which is required to form stable football-type complexes. 

Interestingly, human HSP60 did not bind HSP10 by AMP-PNP although porcine HSP60 bound HSP10 and a protein folding reaction was promoted [20]. This difference may reflect the strong specificity of human HSP60 to ATP, not ADP nor other non-hydrolysable ATP analogues. The folding reaction of porcine HSP60 promoted by AMP-PNP indicates that ATP binding, not ATP hydrolysis, is required for HSP10 binding to HSP60 and subsequent formation of the football complex.

In the presence of ATP and HSP10, HSP60 mainly exists as a double ring and especially half of HSP60 exists as a football-type complex although all of GroEL/GroES forms bullet-type complexes under the same condition. It has been reported that the HSP60/HSP10 complex easily forms a football complex in contrast to the GroEL/GroES complex [30,33,34,35,36,37]. This is because ATP is unable to bind to the trans-ring of the GroEL/GroES bullet when the trans-ring is occupied by ADP, which results in the prevention of football complex formation [35]. However, GroEL/GroES also can be detected as football-type complexes under various non-physiological conditions [38,39,40,41,42,43,44,45]. In addition, substrate protein stimulates the football complex formation of GroEL/GroES by facilitating the dissociation of ADP from the trans-ring and overcoming the inter-ring negative cooperativity [43,44,45]. In contrast, a D398A mutant of GroEL stably forms the football-type complex [31,46,47]. These findings show that the binding of ATP to both rings is important for the formation of football-type complexes for both GroEL/GroES and HSP60/HSP10. 

**Figure 6:**
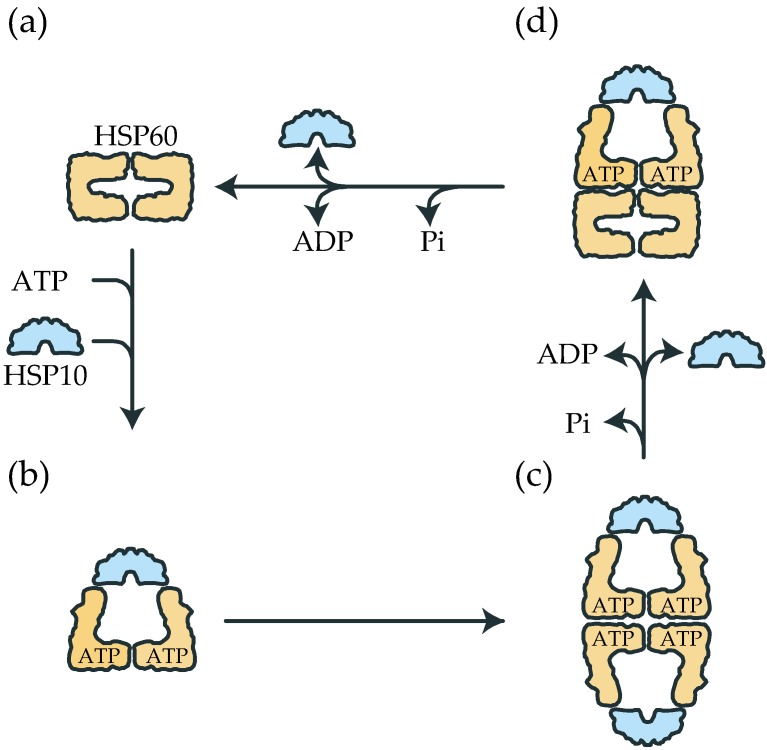
The proposed reaction scheme of human HSP60/HSP10. (**a**) Single-ring state formed by dissociation of HSP10 and ADP after ATP hydrolysis in the bullet-type complex in (**d**). (**b**) The binding of ATP and HSP10 to HSP60 to form single-ring HSP60/HSP10/ATP complex. (**c**) The formation of the double-ring football complex by association of two HSP60/HSP10/ATP single-rings. (**d**) The formation of the bullet-type complex by dissociation of HSP10 and ADP after ATP hydrolysis in one of the rings.

Based on our present data and previous observations [16,17,18,19,20], we propose a major reaction cycle of human HSP60/HSP10 (Figure 6). HSP60 exists as a single ring in the nucleotide-free state (step a). After the ring is occupied by ATP, HSP60 associates with HSP10 (step b). Subsequently, two HSP60 single-rings associate and form a football complex (step c). This phase is predicted to be the longest phase from the population in our TEM and SAXS results. Next, the ATP in one of the two rings is hydrolyzed to ADP, resulting in the release of the HSP10 and ADP and the formation of bullet-type complexes (Step d). Although the nucleotide content in the bullet-type was not confirmed experimentally, the hydrolysis of ATP in two rings would not be synchronized as the case of GroEL [43,45]. The formation of a bullet-type complex from a single-ring/HSP10/ATP complex and single-ring/ATP could be possible. However, in the presence of excess HSP10, this possibility would be rare. Finally, the hydrolysis of ATP in another ring induces the release of GroES and ADP and the subsequent dissociation into two HSP60 single rings. Different from GroEL/GroES, the reaction scheme of the HSP60/HSP10 system includes the transition between double-ring and single-ring. The formation of two folding-competent rings of HSP60/HSP10 may be suitable in mammalian mitochondria in which nascent proteins are constantly produced unlike bacteria which could fall into a resting state by starvation or low temperature.

## 4. Materials and Methods

Plasmid construction-Human HSP60 (without mitochondrial signal sequence) and HSP10 cDNAs (StressGen; San Diego, CA, USA) were amplified by PCR using specific primers. To create a D398A mutant of HSP60 and an A2C mutant of HSP10, the codon for the 398th amino acid of HSP60 was mutated to that of alanine and the codon for the second amino acid of HSP10 was mutated to that of cysteine using QuikChange Site-Directed Mutagenesis Kit (Stratagene, La Jolla, CA, USA). Human HSP60 and HSP10 (wild type and A2C mutant) cDNAs were subcloned into the NdeI/BamHI sites of pET-3a vector (Novagen, Madison, WI, USA) by PCR with their specific primers.

HSP60-*Eschericha coli* BL21 (DE3) pLysS were transfected with the HSP60 expression vector (wild type or D398A mutant), and bacteria were grown in LB medium supplemented with ampicillin (50 μg/mL) and chloramphenicol (34 μg/mL) at 37 °C. Expression of recombinant proteins was induced by addition of 0.1 mM isopropyl-1-thio-β-d-galactopyranoside. Cells were re-suspended in buffer A (1 mM ethylenediaminetetraacetic acid (EDTA), 1 mM dithiothreitol (DTT), 5% Glycerol and 10 mM 4-(2-hydroxyethyl)-1-piperazineethanesulfonic acid (HEPES)-KOH pH 7.4) and lysed by sonication on ice. Supernatants were recovered after centrifugation (20,000 rpm, 15 min) and further fractionated by ammonium sulfate precipitation. Supernatants after 20% ammonium sulfate precipitation were applied to a Butyl 650M-Toyopearl column (Tosoh, Tokyo, Japan) equilibrated with 20% saturated ammonium sulfate in buffer A. After washing with 20% saturated ammonium sulfate and 20% methanol in buffer A, proteins were eluted with a linear gradient of 20–0% saturated ammonium sulfate in buffer A. Proteins in HSP60-rich fractions were recovered by 60% saturated ammonium sulfate precipitation and dissolved in 0.1 M NaCl/buffer A. Proteins were applied onto a Sephacryl S-300 column (GE Healthcare, Uppsala, Sweden) equilibrated with 0.1 M NaCl/buffer A. HSP60 fractions were applied onto Heparin Sepharose 6 Fast Flow column (GE Healthcare) equilibrated with buffer A. After washing with buffer A, proteins were eluted with a linear gradient of 0–0.1 M NaCl in buffer A. After concentration by ultrafiltration, HSP60 fractions were separated by a Superdex 200 10/300 GL column (GE Healthcare) equilibrated with 0.1 M NaCl/buffer A. HSP60 fractions were concentrated, and buffer was exchanged with buffer B (10% Grycerol and 50 mM HEPES-KOH pH7.4). 

HSP10-*Eschericha coli* expressing HSP10 (wild type or A2C mutant) was lysed by sonication, and extracted proteins were treated as described above until 60% saturated ammonium sulfate precipitation was performed. Precipitated proteins were dissolved and dialyzed against buffer C (1 mM EDTA and 10 mM sodium acetate, pH 5.2). After removal of the insoluble fraction by centrifugation (20,000 rpm, 15 min), proteins were applied to a Sulphopropyl (SP) Sepharose Fast Flow column (GE Healthcare) equilibrated with buffer C. After washing with 50 mM NaCl in buffer C, proteins were eluted with a linear gradient of 0–0.7 M NaCl in buffer C. HSP10 fractions were concentrated, and buffer was exchanged with buffer B. 

GroEL and GroES-GroEL and GroES were expressed in *Eschericha coli* AD21 carrying pKY206 groELS plasmid. Cells were grown in Luria broth (LB) medium supplemented with tetracycline (12.5 μg/mL) and 0.2% glucose at 37 °C. Cells were lysed in buffer A by sonication, and extracted proteins were treated as described above until 60% saturated ammonium sulfate precipitation was performed. After proteins were dissolved and dialyzed against buffer C, protein samples were applied onto a Sephacryl S-300 column equilibrated with 0.1 M NaCl/buffer A. GroEL fractions were concentrated, and buffer was exchanged with buffer B. GroES rich fractions were dialyzed against buffer C, and the insoluble fraction was removed by centrifugation (20,000 rpm, 15 min). Proteins were applied to SP Sepharose Fast Flow column equilibrated with buffer C. After washing with 50 mM NaCl in buffer C, proteins were eluted with a linear gradient of 0–0.7 M NaCl in buffer C. GroES fractions were concentrated, and buffer was exchanged with buffer B.

Size exclusion chromatography-HSP60 and GroEL (0.41 μM) were analyzed by size exclusion chromatography on a Superdex 200 10/300 GL column equilibrated with 0.1 M NaCl, 5% grycerol and 50 mM HEPES-KOH (pH7.4). Protein concentration was monitored by absorption at 280 nm. Calibration curve was obtained by plotting of logalism of molecular weight of control samples versus their elution volume.

Transmission electron microscopy-HSP60 (0.1 μM)/HSP10 (0.2 μM), HSP60 (0.1 μM), or GroEL (0.1 μM)/GroES (0.2 μM) were incubated in the presence of or absence of 1 mM nucleotides (ATP, ADP, ATPγS or AMP-PNP) in buffer D (10 mM MgCl_2_, 20 mM KCl, 1 mM DTT and 50 mM HEPES-KOH pH 7.4) at 25°C for 10 min. After incubation, these samples were treated with 0.2% uranyl acetate and analyzed by transmission electron microscopy (H-7650, Hitachi, Tokyo, Japan). Chaperonin molecule classifications were performed by manually counting these molecules three times, and more than 500 molecule were counted for each counting experiment. 

SAXS-All SAXS data were obtained at the BL40B2 beam line in the SPring-8 synchrotron radiation facility (Hyogo, Japan) at 25°C. Scattering profiles were collected by a detector system using R-axis VII imaging plates (Rigaku, Tokyo, Japan). X-ray wavelength and camera length were 1.000 Å and 2116 mm, respectively. HSP60 or GroEL (3.0 mg/mL) were incubated with various concentrations of co-chaperones and 1 mM nucleotides or without these materials in buffer D, and measurements were performed using a sample cell of 3 mm path length. The data collection times were 60 s, and no changes were observed in SAXS profiles by sequential X-ray exposures. The radius of gyration (*R*_g_) and *I*(0) were calculated from the Guinier approximation using the innermost portion of the scattering patterns; the slope and the intercept of the semilogarithmic plot (the Guinier plot) of ln *I*(Q) versus Q2 gave the *R*_g_ and *I*(0), respectively, where *I*(Q) is forward scattering intensity as a function of the momentum transfer Q = 4πsinθ/λ, and 2θ is the scattering angle. *I*(0)/C gave an estimate of apparent molecular mass of solute, where C is the protein concentration of HSP60 or GroEL. After a circular average of the two-dimensional data and buffer subtractions, the pair distribution function *P*(r) was calculated using the GNOM program [48]. The maximum particle distance (*D*_max_) was calculated from the *P*(r) function.

Protease Sensitivity Assay-HSP60/HSP10 or GroEL/GroES (1 μM) were preincubated with various nucleotides (1 mM) at 25 °C for 10 min. Protein samples were digested by trypsin (10 mg/mL) at 25 °C for 15 min. Digestion reaction was stopped by adding Sodium dodecyl sulfate (SDS) sample buffer and heating at 100 °C for 3 min. Digested proteins were separated by SDS-PAGE (12% gel) and detected by Coomassie Brilliant Blue R-250 staining. Amount of Trypsin-resistant HSP10 and GroES was quantified by the Image J program (National Institute of Health, Bethesda, MD, USA) after scanning gel images.

Fluorescence labeling of proteins-Fluorescence labeling was performed based on the manufacturer’s instructions. HSP60 and HSP10-A2C (50 nmol, 50 nmol) were labeled using Atto 647N maleimide and Atto 488 maleimide (15 nmol, 50 nmol, 4 °C, 16 h) (Fluka analytical, Buchs, Switzerland), respectively. After buffer was exchanged with buffer B, labeled proteins were concentrated by ultrafiltration. Concentrated proteins were applied onto a Superdex 200 10/300 GL column equilibrated with 0.1 M NaCl/buffer A. Fractions containing labeled proteins were concentrated, and buffer was exchanged with buffer B. Molar extinction coefficients were calculated in ProtoPramtool in ExPASy Proteomics Server (Available online: http://kr.expasy.org/ tools/protparam.htmL). Yield of fluorescence labeling was spectrophotometrically determined using molecular extinction coefficients of ε644 (Atto 647N) = 150,000 cm^−1^·M^−1^ and ε501 (Atto 488) = 68,000 cm^−1^·M^−1^. 

FCCS measurement-FCCS measurement was performed on a ConfoCor2 system (Carl Zeiss, Jena, Germany) essentially as described previously20. Atto 647N-labeled HSP60 (0.4 μM) and Atto 488-labeled HSP10-A2C (8 μM) were immediately mixed in buffer E (10 mM MgCl_2_, 20 mM KCl and 50 mM HEPES-KOH, pH 7.4) supplemented with various nucleotides (1 mM). Samples were applied to a Lab-Tek 8-well chamber (Nalge Nunc, Naperville, IL, USA) coated with bovine serum albumin. These samples were simultaneously excited by two laser beams at 488 nm and 633 nm and measured at 25 °C. To obtain, the number of fluorescent molecules, we fitted auto- and cross-correlation functions using one- or two-component diffusion models using the AIM version 3.2 software (Carl Zeiss, Jena, Germany). The relative cross correlation amplitudes (RCA) were calculated by (Number of cross correlated molecule)/(Number of HSP60 molecule) × 100, and values of a binding rate were calculated by (RCA of sample)/(RCA of positive control) × 100. Dissociation constant (*K*_d_) values were calculated by the equations as follows, and the volume overlapped by two excitation beams (*V*_gr_) was defined as (*V*_r_) × (RCA of positive control) as follows, and the volume overlapped by two excitation beams (*V*_gr_) was defined as (*V*_r_) × (RCA of positive control).

$$K_{d}=\frac{\left(\mathrm{HSP}10_{\mathrm{free}}\right)}{\left(\mathrm{Complex}\right)}$$

For determination of maximum value of RCA, HSP60 was labeled with both Atto 488 and Atto 647N as a positive control (Figure S5A). For determination of minimum value of RCA, a mixture of fluorescent probes (Atto 488 and Atto 647N) was used as a negative control (Figure S5B) and the RCA value was observed as 0.00.

ATPase assay-For high pressure liquid chromatography (HPLC) analysis, purified chaperonins or co-chaperon (0.1 μM) were incubated with nucleotide (1 μM) at 37°C. Nucleotides were separated using C18 reverse phase column (Mightysil PR-18CP, 4.6 mm ID × 150 mm, Kanto Kagaku: Tokyo, Japan) chromatography using HPLC. The liquid chromatographic equipment consisted of a PU-1580 intelligent HPLC pump, LG-1580-02 ternary gradient unit (Jasco: Tokyo, Japan), SPD6A spectrophotometric detector, and CTO6A column oven (Shimadzu, Kyoto, Japan). Data were recorded and analyzed with a LabVIEW software system Version 7.1 (National Instruments, Austin, TX, USA). Chromatographic determination was performed at a flow rate of 1 mL/min at 37 °C, and the detection wavelength was set at 256 nm [49].

Native PAGE-Chaperonins (0.1 μM) were incubated in an assay buffer D in the absence or presence of ATP (1 mM ATP) and co-chaperones (0.1 μM) at 25 °C for 10 min. Samples were electrophoresed on 5% acrylamide gels in the absence or presence of 1 mM ATP in native-PAGE buffer (50 mM Tris-400 mM glycine). 

Rhodanese folding assay-Rhodanese (5 μM) was denatured at 25 °C for 60 min in denaturing buffer (50 mM HEPES-KOH, pH 7.4, 1 mM EDTA, and 1 mM DTT, 6 M guanidine hydrochloride). Denatured rhodanese was diluted 200 times in refolding buffer (50 mM HEPES-KOH, pH 7.4, 10 mM MgCl_2_, 20 mM KCl, 20 mM Na_2_S_2_O_3_, 200 mM glucose and 1 mM DTT) containing 0.2 µM chaperonin (HSP60 or GroEL) and 0.4 µM co-chaperone (HSP10 or GroES). Refolding assay was started by an addition of 1 mM ATP. For single turnover ATP-hydrolysis experiments, the excess ATP was hydrolyzed to ADP by adding hexokinase (final concentration 0.08 units/µL) to the reaction mixture at 3 s after the initiation of reaction. At the times indicated, the folding reaction was stopped by mixing aliquots of the solution containing 67 mM KH_2_PO_4_, 83 mM Na_2_S_2_O_3_, 10 mM EDTA and 0.1 mg/mL Bovine serum albumin (BSA) on ice. The enzymatic assay was initiated by adding 0.25 M KCN at 25 °C and the enzymatic reaction was terminated by adding 37% formaldehyde after 30 min. After addition of ferric nitrate reagent, the rhodanese activity was measured colorimetrically by the absorbance at 460 nm.

## Figures and Tables

**Table 1:** Relative cross correlation amplitude (RCA) and dissociation constant (*K*_d_) of HSP60/HSP10 complex calculated from Fluorescence cross correlation spectroscopy (FCCS) analysis.

Nucleotide	RCA	Binding Ratio (%)	*K*_d_ (M)
Free	0.22	35.1	7.5 × 10^−7^
ATP	0.55	86.8	8.7 × 10^−8^
ADP	0.25	40.2	5.1 × 10^−7^
AMP-PNP	0.20	32.5	7.9 × 10^−7^
ATPγS	0.23	36.6	7.2 × 10^−7^

Binding ratio was estimated using a positive control (double-labeled molecule; set to 100%) and a negative control (two free dyes; 0%). Abbreviation: adenosine triphosphate (ATP), adenosine diphosphate (ADP), Adenosine-5′-(β, γ-imido)-triphosphate (AMP-PNP) and adenosine 5′-(γ-thio)-triphosphate (ATPγS)

## Supplementary Materials

Supplementary materials can be found at http://www.mdpi.com/1422-0067/19/2/489/s1.
