# Supplementary material for: Physicochemical Properties of the Mammalian Molecular Chaperone HSP60
Source: Int J Mol Sci. 2018 Feb 6;19(2):489. doi: 10.3390/ijms19020489 (PMC5855711; doi:10.3390/ijms19020489)
Supplement: Supplementary file 1 [file ijms-19-00489-s001.pdf]

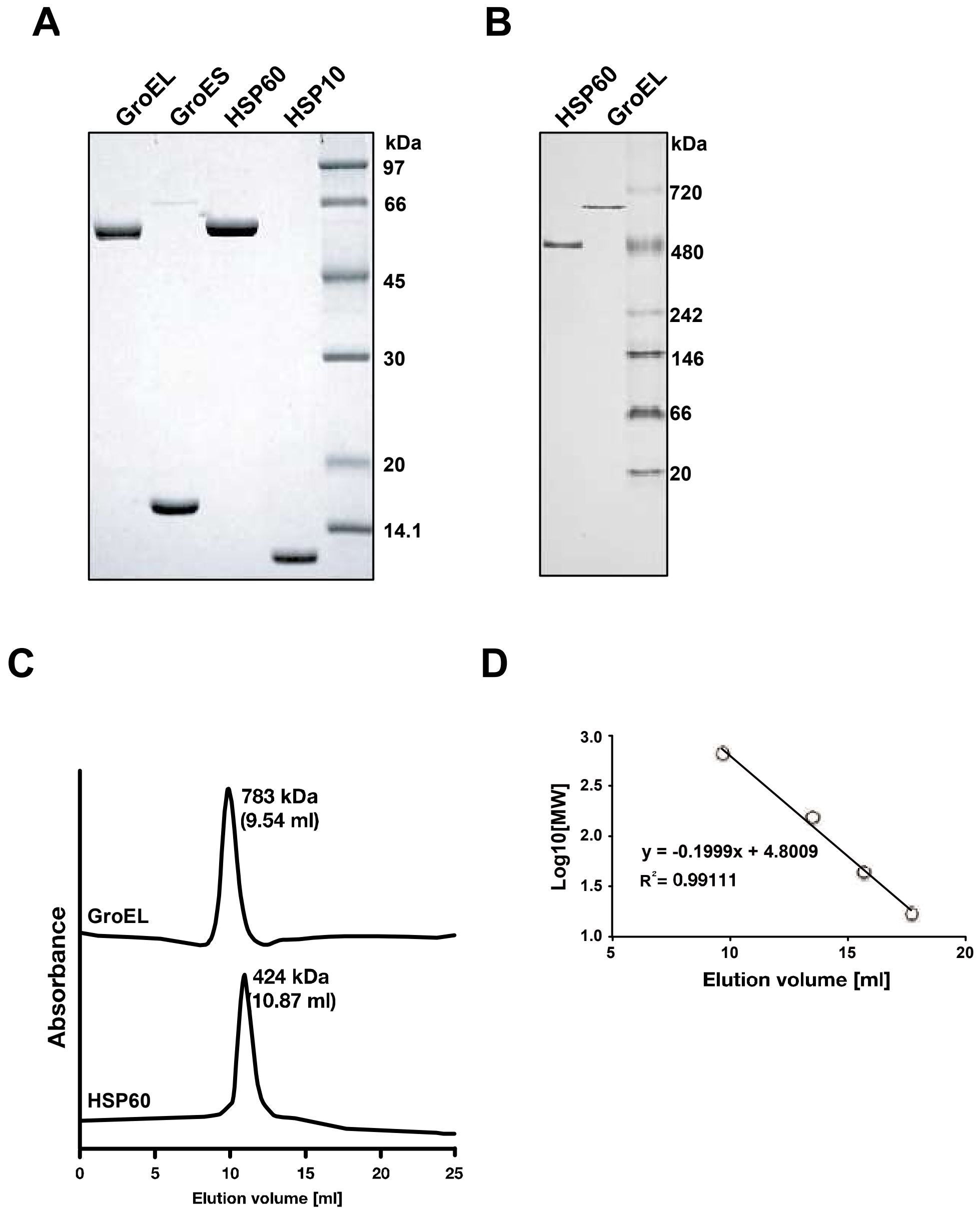

Supplemental Figure S1. Molecular size of HSP60 in the presence or absence of co-chaperone HSP10. (A) SDS-PAGE analysis (12% gel) of purified chaperonins. **(B)** Native-PAGE analysis (5-12% gradient gel) of HSP60 and GroEL in the absence of nucleotides. **(C)** Size exclusion chromatography of HSP60 and GroEL in the absence of nucleotides. **(D)** Calibration plot of the logalism of molecular weight of control samples versus elution volume for size exclusion chromatography.

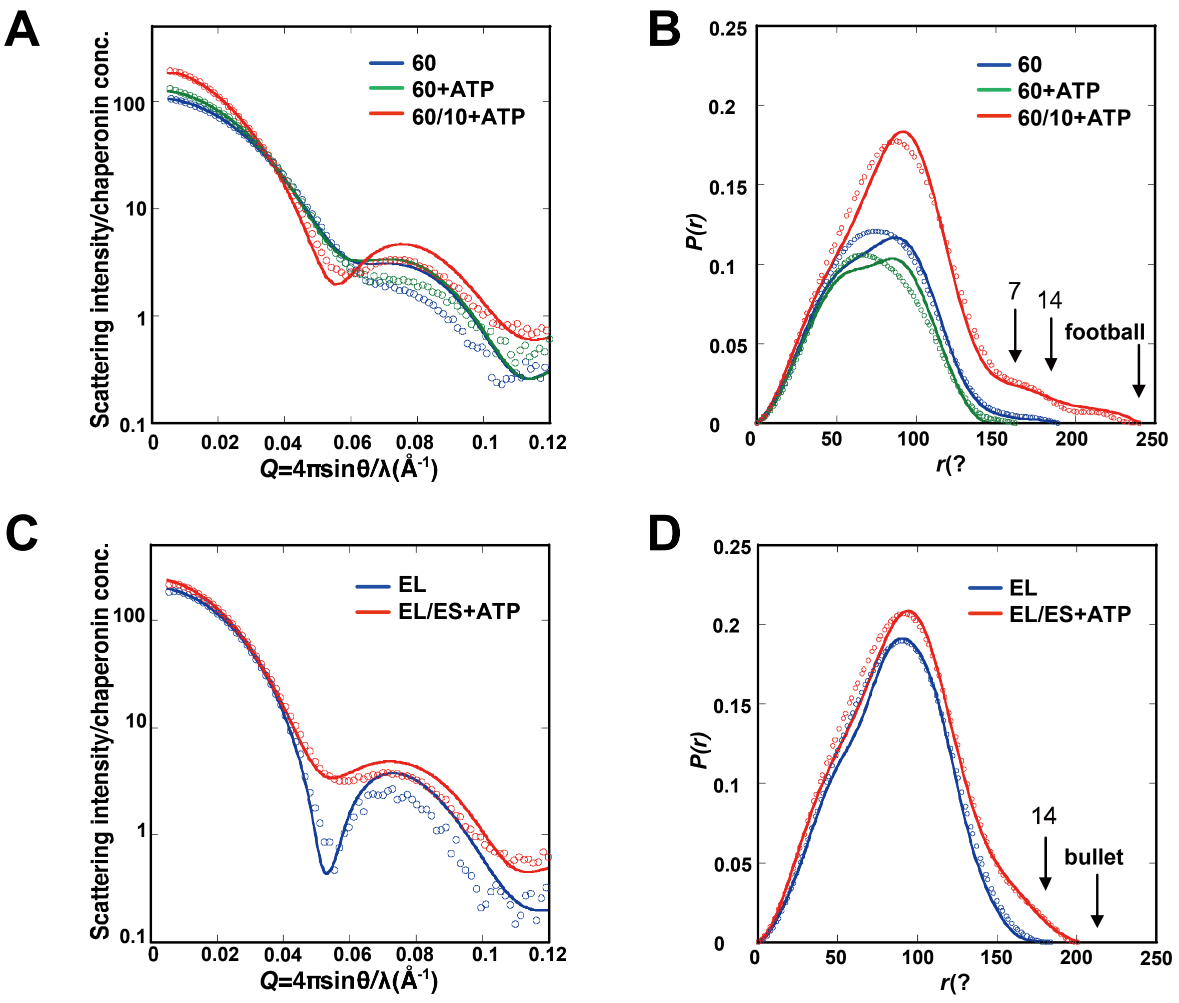

Supplemental Figure S2. SAXS analysis of HSP60/HSP10 and GroEL/GroES complex in the various nucleotide conditions. The observed data were shown in open circles. The fitting curves calculated by CRYSOL or OLIGOMER were indicated as solid [47]. The theoretical D_{max} calculated from crystal structures of single-ring of GroEL (PDB: 1OEL, D_{max}=167 Å), double-ring of GroEL (PDB: 1OEL, D_{max}=186 Å), bullet-type complex of GroEL/GroES/ADP (PDB: 1AON, D_{max}=216 Å) and football-type complex of human HSP60(E321K)/HSP10/ADP (PDB: 4PJ1, D_{max}=245 Å) are indicated by arrows and labeled with 7, 14, bullet, and football, respectively. **(A, B)** SAXS patterns **(A)** and the P(r) function **(B)** of HSP60 (blue), HSP60/ATP (green), and HSP60/Hsp10+ATP (red). SAXS pattern of HSP60 was fitted with the theoretical scattering curve of the single-ring GroEL. SAXS pattern of HSP60/ATP was fitted with the theoretical model of single-ring and double-ring GroEL in HSP60+ATP at the ratio of 0.86 and 0.14, respectively. In the presence of HSP10 and ATP, the scattering pattern was largely changed and fitted with the theoretical curves of single-ring GroEL, single-ring HSP60/HSP10 complex, and the football complex at the mixture ratio of 0.09, 0.57, and 0.34, respectively. **(C, D)** SAXS patterns **(C)** and the P(r) function **(D)** of GroEL (blue) and GroEL/GroES+ATP (red). SAXS pattern of GroEL was fitted with the theoretical curve of double-ring GroEL. SAXS pattern of GroEL/GroES+ATP was fitted with the theoretical curve of bullet-type complex and GroES at the ratio of 0.75 and 0.25, respectively.

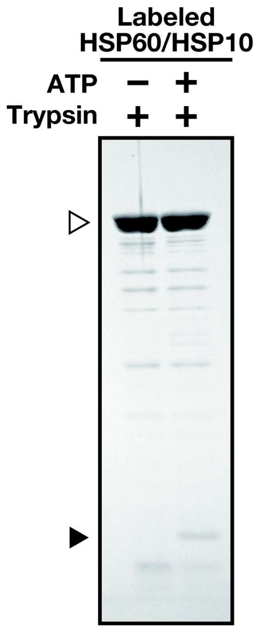

Supplemental Figure S3. Protease sensitivity assay to measure association of Atto 488 labeled HSP10-A2C to Atto 647N-labeled HSP60 in the presence of ATP. Experiment was performed as Fig. 3A using labeled samples.

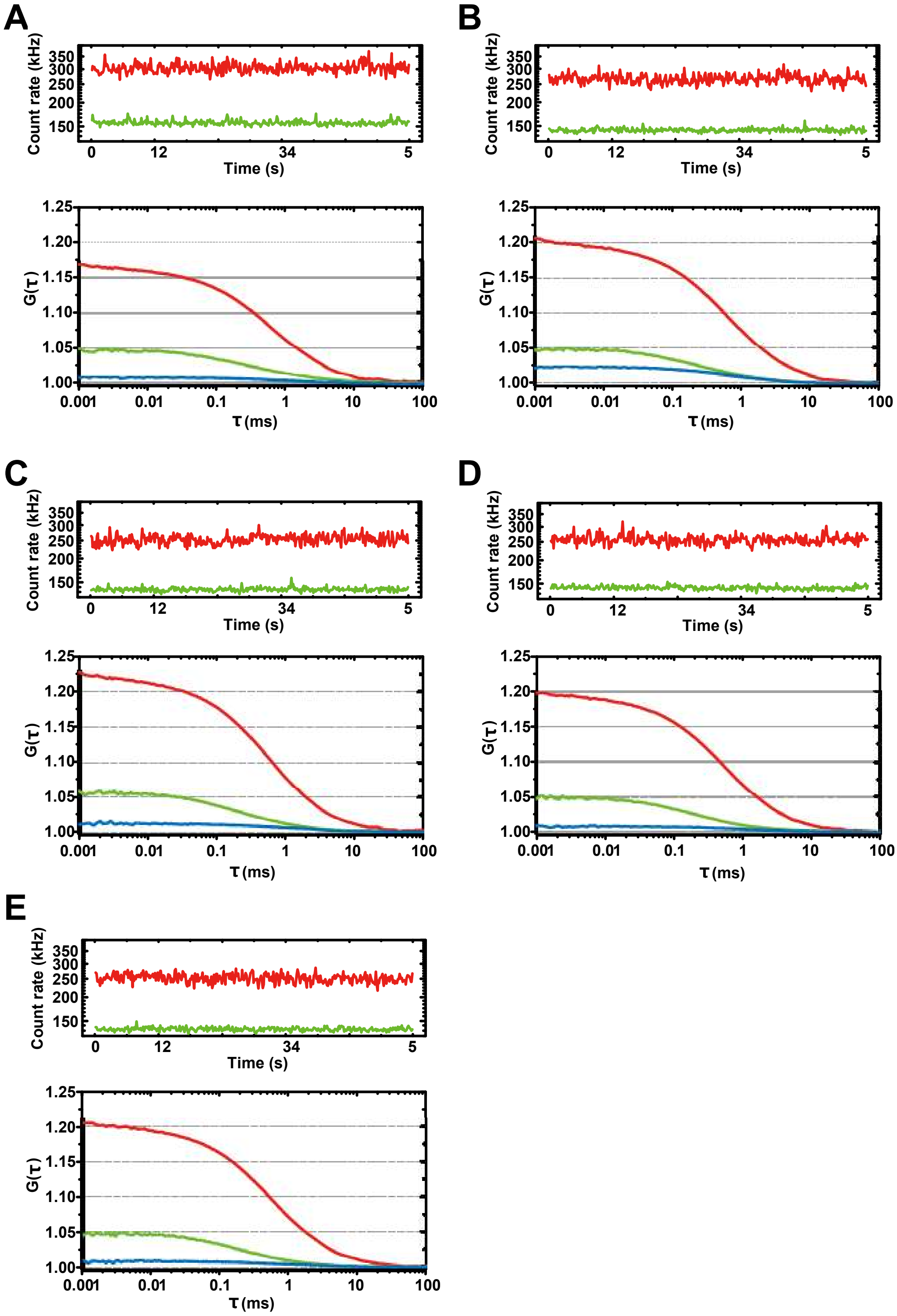

Supplemental Fig. S4. FCCS analysis of the association between HSP60 and HSP10. Measured fluorescence fluctuations (upper) and calculated auto-correlation functions (lower) of Atto 647N-labeled HSP60 (red line) and Atto 488-labeled HSP10 (green line). Cross-correlation functions between green and red fluorescence detection channels are shown as blue lines. In the absence of nucleotide **(A)**, or in the presence of 1 mM ATP **(B)**, ADP **(C)**, AMP-PNP **(D)**, or ATPγS **(E)** were shown.

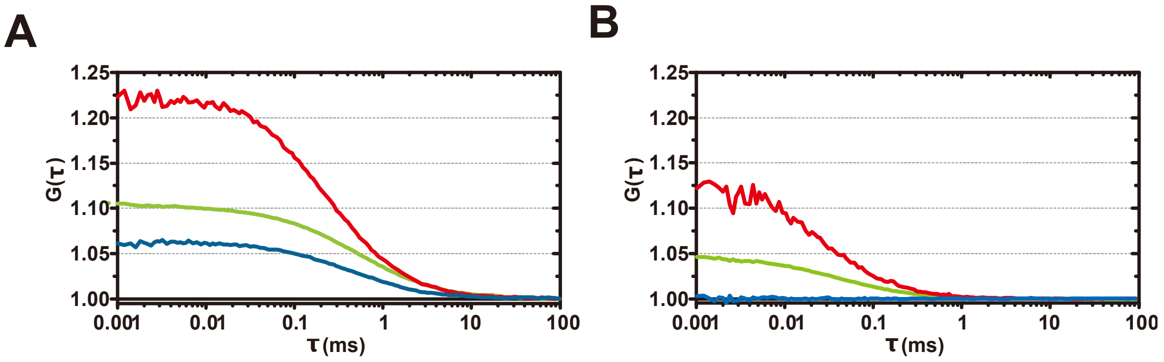

Supplemental Fig. S5. FCCS analysis of Atto 488/Atto 647N double labeled HSP60 as positive control (A) and that of two dyes as negative control (B). The calculated auto-correlation functions of Atto 647N and Atto 488 were shown as red lines and green lines, respectively. Cross-correlation functions are shown as blue lines.

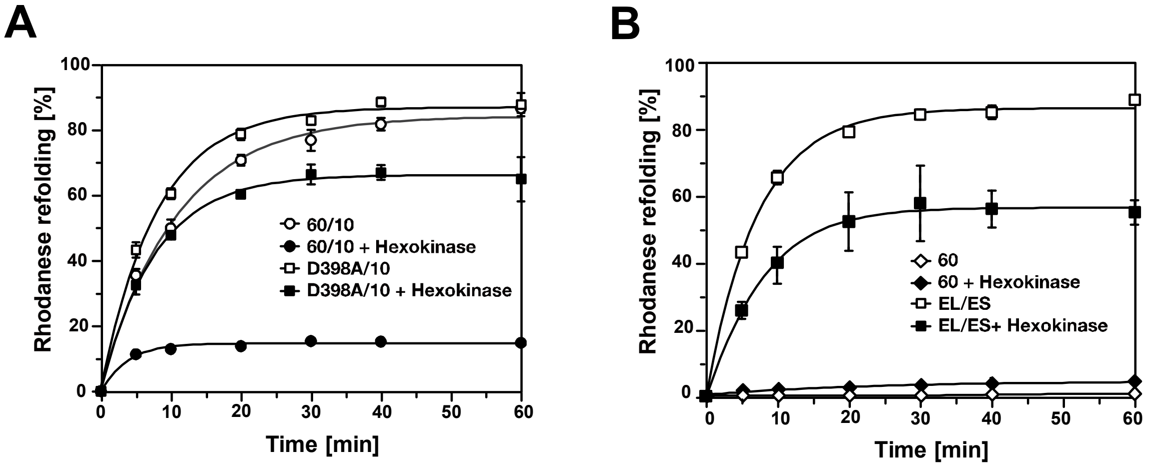

Supplemental Fig. S6. Time course of rhodanese refolding by HSP60/HSP10 and HSP60(D398A)/HSP10. (A) Recovered rhodanese activity by wild type HSP60 (circle) and HSP60(D398A) (square) with (closed) or without (open) addition of hexokinase at 3 s after the initiation of refolding reaction (n=3). **(B)** Recovered rhodanese activity by HSP60 (diamond) or GroEL/GroES (square) with (closed) or without (open) hexokinase treatment. (n=3).
